# Facilitators of health systems change for tobacco dependence treatment: a qualitative study of stakeholders’ perceptions

**DOI:** 10.1186/s12913-014-0575-4

**Published:** 2014-11-19

**Authors:** Amanda L Jansen, Traci R Capesius, Randi Lachter, Lija O Greenseid, Paula A Keller

**Affiliations:** ClearWay MinnesotaSM, Minneapolis, USA; Professional Data Analysts, Inc., Minneapolis, USA

**Keywords:** Tobacco, Systems change, Quality improvement, Electronic health records, Process evaluation

## Abstract

**Background:**

Health systems play key roles in identifying tobacco users and providing evidence-based care to help them quit. Health systems change – changes to health care processes, policies and financing – has potential to build capacity within these systems to address tobacco use. In 2010, ClearWay Minnesota^SM^ piloted a health systems change funding initiative, providing resources and technical assistance to four health care systems. This paper presents findings from a process evaluation, describing key stakeholders’ views on whether changes to how health systems treat tobacco use resulted from this initiative and what may have facilitated those changes.

**Methods:**

A process evaluation was conducted by an independent evaluation firm. A qualitative case study approach provided understanding of systems change efforts. Interviews were conducted with key informants representing the health systems, funder and technical assistance providers. Core documents were reviewed and compared to thematic analysis from the interviews. Results were triangulated with existing literature to check for convergence or divergence. A cross-case analysis of the findings was conducted in which themes were compared and contrasted.

**Results:**

All systems created and implemented well-defined written tobacco use screening, documentation and treatment referral protocols for every patient at every visit. Three implemented systematic follow-up procedures for patients referred to treatment, and three also implemented changes to electronic health records systems to facilitate screening, referral and reporting. Fax referral to quitline services was implemented or enhanced by two systems. Elements that facilitated successful systems changes included capitalizing on environmental changes, ensuring participation and support at all organizational levels, using technology, establishing ongoing training and continuous quality improvement mechanisms and leveraging external funding and technical assistance.

**Conclusions:**

This evaluation demonstrates that health systems can implement substantial changes to facilitate routine treatment of tobacco dependence in a relatively short timeframe. Implementing best practices like these, including increased emphasis on the implementation and use of electronic health record systems and healthcare quality measures, is increasingly important given the changing health care environment. Lessons learned from this project can be resources for states and health systems likely to implement similar systems changes.

## Background

Tobacco dependence is recognized as a chronic, relapsing condition that requires ongoing evaluation and, as appropriate, intervention to support quitting and abstinence [1]. The most recent report of the Surgeon General states that “the current rate of progress in tobacco control is not fast enough, and much more needs to be done to end the tobacco epidemic [2]”. Since 70 percent of smokers see a clinician in a given year, health systems can play a key role in identifying tobacco users and providing and referring them to evidence-based quitting help [1]. However, research shows substantial room for improvement in integrating comprehensive tobacco dependence treatment into routine health care. For example, data from Minnesota show that while 94 percent of smokers seen by providers in the past 12 months reported they were asked about smoking, fewer than half reported receiving treatment or referrals to quitting assistance [3]. Moreover, data demonstrate that treating tobacco use leads to higher patient satisfaction [4], improved patient health [5] and a positive return on investment [6], making treating tobacco use an ideal target for health care systems change efforts.

Health care systems changes – changes to health care processes, policies and financing – can result in tobacco use being addressed routinely with all patients in an ongoing, sustainable manner [7]. The U.S. Centers for Disease Control and Prevention (CDC) has recommended the implementation of health systems change in its recent update of Best Practices for Comprehensive Tobacco Control Programs [8].

There is a robust body of research detailing the effectiveness of systems change strategies, including tobacco user identification systems, provider training and feedback [1,9-11]. Using electronic health records (EHRs) for tobacco user identification and subsequent interventions also increases delivery of evidence-based care [12,13]. Additionally, linking EHR documentation or tobacco user identification systems to quality improvement and provider feedback mechanisms improves adherence to best practices, including documentation of tobacco use status and cessation service referral [14-17]. However, success requires more than just implementation of these strategies; leadership and process improvement support are also required to help create a culture where routinely addressing tobacco use is the norm [18,19]. As the health care environment moves toward widespread adoption of EHRs, and payers are providing financial incentives for their adoption and use (e.g., Meaningful Use), there is a growing potential to integrate evidence-based tobacco dependence treatment into existing health care. There is also increased visibility of health systems change, with its incorporation by the CDC as a best practice [8]. In this paper, we examine factors that influenced health systems’ ability to implement systems changes, including making changes with their EHRs.

ClearWay Minnesota^SM^, an independent nonprofit funded with 3 percent of Minnesota’s settlement with the tobacco industry, has a longstanding interest in identifying sustainable approaches to reducing the harm that tobacco causes Minnesotans. In 2010, ClearWay Minnesota funded a 21-month health systems change initiative, providing resources and technical assistance to four health care systems that had previously been funded to provide intensive cessation services.

### Setting

Four Minnesota health systems were funded to implement health systems changes. Health System 1 was a large, primarily rural, integrated health system that included hospitals, clinics, long-term care facilities, assisted living facilities, independent living facilities and a research institute. Their systems change initiative was focused on a few primary care clinics within this larger health system. Health System 2, also a large, integrated health system, focused on six rural primary care clinic sites for this initiative. Health System 3 was a multi-specialty, teaching-focused outpatient care facility located in an urban area. This initiative was focused on four residency clinic sites within this larger system. Health System 4 was an urban, federally-funded community health center that provides primary care, dental care, behavioral health care, and chiropractic and eye care via its two clinic sites. Both clinics were the focus of this systems change initiative. Each system was required to choose systems change strategies from a provided list to guide their work (see Table 1, Systems Change Strategies Chosen by Each System). The list of strategies was derived from the published literature [1] and from health systems change experts. Health systems were deemed successful if they were able to make operational changes positioning them to address tobacco dependence more consistently and effectively.

**Table 1:** **Systems change strategies chosen by each system**

**Systems change strategies**	**System 1**	**System 2**	**System 3**	**System 4**
Identification and documentation (required): Implement a clinic-wide system that ensures for every patient at every clinic visit, tobacco-use status is queried and documented.	X	X	X	X
Training and education: provide adequate training, resources and education to motivate clinic personnel to address tobacco and increase tobacco treatment knowledge and skills.	X		X	X
Referrals: establish a referral system for tobacco users interested in counseling. Referrals can be internal and/or to external, evidence-based cessation services as is most appropriate for the patient population being served by the system.	X	X	X	X
Brief interventions: Institutionalize brief tobacco cessation interventions into clinic protocols.				
Reporting and feedback: Establish a reporting and feedback system that facilitates regular retrieval of and consistent feedback on tobacco use and treatment data to clinic personnel and that can be used to track tobacco-related performance measures and quality improvement initiatives.	X		X	
Follow up care: systematically follow up with patients who receive brief tobacco cessation interventions or referral to cessation services to determine actual provision of services and ongoing needs.	X		X	
System integration: Integrate tobacco cessation approaches into disease management protocols.	X		X	
Quality improvement, Accreditation and Standards: Systematically integrate tobacco cessation strategies into existing programs or initiatives that address quality improvement, accreditation and evaluative standards in a way that will lead to improvements in the use of selected cessation strategies.			X	
Organizational goals and policies: Ensure that organizational goals and policies related to tobacco cessation are consistent and supportive of systems change strategies and activities.		X		
Hospital policies and services: Implement hospital policies that support and facilitate inpatient tobacco dependence services.				
Performance measures and follow up: provide regular feedback to clinic personnel to ensure providers consistently deliver effective tobacco cessation treatment or referrals as per the established protocol.	X	X	X	
Reimbursement: if relevant, establish a system to consistently seek and obtain reimbursement for tobacco cessation services.	X			
Tobacco cessation coverage: Improve patient education about and use of existing cessation benefits (counseling and medication).		X		

As part of the funding opportunity, health systems were also provided with technical assistance (from program staff and a contracted technical assistance provider) to support them in implementing their systems change strategies. Technical assistance was funded by ClearWay Minnesota and included, but was not limited to, implementing an effective tobacco-user identification system, educating about effective uses of an electronic health record (EHR) to enhance tobacco cessation interventions, and training and educating providers. Technical assistance was provided to each project by phone, by email and in person.

This work was evaluated to gauge whether such an initiative could result in changes that impact how health systems treat tobacco use. Moreover, this evaluation sought to identify factors that facilitated implementation of health systems change, and to better understand whether and how EHRs could be used to drive or support these types of change. This paper summarizes findings from this evaluation.

## Methods

An independent process evaluation, funded by ClearWay Minnesota, was conducted by Professional Data Analysts, Inc., a research and evaluation firm specializing in tobacco control evaluation. A qualitative case study approach was used to gain an in-depth perspective [20] on the successes, challenges, lessons learned and potential sustainability of the tobacco-related systems changes implemented by each health system. Qualitative research methods, such as those employed in this evaluation, are appropriate for studies seeking deep understanding of complex issues that cannot be measured by existing standardized metrics [21]. Two complementary data methods were used: a core document review and key informant interviews. Results from the document analysis and key informant interviews were triangulated with existing literature to check for convergence or divergence from existing findings in the field of tobacco cessation systems change research. A cross-case analysis of the findings was then conducted, in which themes were compared and contrasted across cases. This study uses information from an administratively driven process evaluation; therefore, human subjects review was not sought.

### Core document review data sources and analysis methods

ClearWay Minnesota provided the evaluators with a set of core documents for each health system. These included quarterly and final progress reports, notes from technical assistance sessions and meetings with ClearWay Minnesota staff, and standardized assessments of capacity to address tobacco use (completed by each health system prior to and at the end of the funding period).

The evaluators reviewed all of the documents to verify progress reported by the grantees during interviews and to assess reported changes pre- and post-initiative. Two evaluators independently documented changes reported by each system and compared the findings to reach agreement on systems changes made during the funding period, as well as to compare and contrast themes that emerged in the analysis of key informant interview data.

### Key informant interview methods and analyses

The evaluators conducted 12 in-depth interviews with a total of 18 key informants representing the health systems, ClearWay Minnesota and technical assistance providers toward the end of the grant cycle. All interviewees consented to have their interviews recorded.

Interview protocols were developed based on an analytic framework suggested by a tobacco systems change literature review and by interviews of experts in the field. The evaluators reviewed documentation received from ClearWay Minnesota to refine interview protocols. The interviews were used to gather additional information on the current state of systems change activities and to document how and the extent to which each site was able to implement chosen strategies.

#### Health system interviews

The evaluators, technical assistance providers and ClearWay Minnesota staff worked together to identify a sample of individuals from each of the health systems who were most knowledgeable about the initiative (e.g., project managers) as well as other key stakeholders who might have different perspectives on the project. Recruitment of interviewees was conducted by email and telephone. Semi-structured interviews with key informants were conducted, toward the end of the 21-month grant funding period. All but one of the interviews were conducted face to face by two evaluators. A minimum of two key informants from each system were interviewed (see Table 2, Health System Key Informants) for a total of 13 health system interviewees. Interviewees from the health systems were asked about key project successes, implementation barriers, lessons learned and advice for other health systems, as well as their perspectives on the potential sustainability of the systems changes they implemented. Additionally, interviewees were asked to provide feedback on the technical assistance they received, as well as to reflect on how the grant helped facilitate systems changes.

**Table 2:** **Health system key informants**

**Health system**	**Interviewee description**	**Background/discipline**
#1: Part of a large, integrated health system; rural and urban populations	Systems change grant project manager	Registered Nurse (RN)
	Tobacco cessation program manager	Nurse practitioner, tobacco treatment specialist (TTS)
	Leadership, champion	Department director
#2: Part of a large, integrated health system; rural and urban populations	Systems change grant project manager	Quality improvement
	Electronic medical records specialist	EMR builder/Information technology
	TTS	Staff member at affiliated hospital
	Physician champion, tobacco cessation	Psychiatry
	TTS	RN
#3: Multi-specialty, teaching-focused out-patient care facilities (four clinic sites); mostly urban population	Systems change grant project manager, clinic project manager	Psychology
	Clinic Project Manager*	Psychology
#4: Federally-qualified health center (two clinic sites); mostly urban population	Systems change grant project manager	Health programs manager
	CEO	Management, quality assurance
	Medical director	Medical provider (MD)

*This was the only grantee interview conducted with only one evaluator present. All other interviews were conducted by two evaluators.

#### ClearWay Minnesota

Two ClearWay Minnesota staff members and an independent contractor hired by ClearWay Minnesota to assist with the project were chosen to participate in the interviews. These interviewees were chosen based on their management and oversight of the grantees as well as on their knowledge of how and why the funding initiative was originally conceived. Semi-structured interviews were conducted face to face by two evaluators immediately after the grant period ended. All three interviewees were asked about key initiative accomplishments; perceptions of critical elements for success, challenges and barriers; and lessons learned and advice for funders considering similar initiatives.

#### Technical assistance providers

Two members of the technical assistance provider team were interviewed by both evaluators – one face to face and the other by telephone immediately after the grant period ended. They were asked about key initiative accomplishments; perceptions of critical elements for success, challenges and barriers; and lessons learned and advice for other technical assistance providers.

### Interview analyses

Summaries of each interview were created by the two evaluators who conducted them. Each summary was provided to interviewees to ensure completeness and accuracy. Corrections or additions from interviewees were incorporated into the final summaries. Evaluators used both deductive and inductive approaches to identify themes. Previous literature provided an initial framework for themes that might arise during cross-case qualitative analyses. Evaluators also identified new themes from the data using an inductive approach. The lead evaluator verified and identified themes based on the literature and interview summaries and by comparing and contrasting the experiences of the four systems. These themes were then reviewed by the second evaluator. Any new themes or differences in interpretation were discussed by evaluators until consensus was achieved. Quotations from interview notes and recordings were de-identified to ensure the confidentiality of the individual and the health system.

## Results

All four systems created and implemented well-defined written protocols to screen for and document tobacco use status for every patient at every visit and to refer tobacco users for tobacco dependence treatment, which was a key success metric for this initiative. Three systems implemented systematic follow-up procedures for patients referred to treatment and three systems also implemented changes to their electronic health records systems to facilitate screening, referral and reporting. Fax referral to quitline services was implemented or enhanced by two systems. This funding initiative, including technical assistance, also played a role in the successful prioritization and implementation of systems change activities.

The evaluation identified five elements that successfully facilitated tobacco-related systems change: 1) capitalize on changes in the environment; 2) ensure participation and support for the project at all levels of the organization; 3) use technology; 4) establish ongoing training and continuous quality improvement mechanisms; and 5) leverage external funding and technical assistance.

### Capitalize on existing quality improvement initiatives

*Respecting competing priorities and taking advantage of existing initiatives can help raise the priority of tobacco dependence treatment.* All systems discussed the difficulty of establishing and maintaining momentum for systems change efforts amid competing priorities. However, all were able to link tobacco treatment to internal and external quality improvement initiatives already being implemented, such as Meaningful Use and Minnesota’s mandatory Statewide Quality Reporting and Measurement System. The existence of tobacco use and treatment components within these measurement systems helped raise the priority of their proposed tobacco-specific systems changes. One system reported, “Smoking cessation is a metric on every quality [care] profile that we’re measured by for reimbursement. . . . This is a standard we’re held to. . . .That [standard] drove us in the beginning and it continues to drive us as we move forward”. Systems change teams demonstrated to providers and administrators how treatment of tobacco dependence would help improve quality measures of greatest concern to them, such as asthma, diabetes or heart disease, and would ultimately help reduce costs associated with tobacco-related illnesses. Another system integrated tobacco use measures into routinely-monitored clinical dashboards for several chronic diseases.

### Ensure participation and support at all levels

*Support from health system leadership is essential.* All health systems were able to garner support from top-level executives and foster champions in key leadership positions (e.g., medical directors, CEOs). One project manager said, “Find your champions early and make sure they are committed, especially if it’s going to involve staff that they supervise”. This support helped free up staff time and resources to develop, pilot and implement these changes. Within one system, meetings were held monthly during the first year and then quarterly with a team consisting of the clinic manager, director of the center (also a physician) and the vice president of operations. These executives reviewed plans, offered suggestions and gave their full support to their systems change endeavors. When the clinical manager left unexpectedly, the center director assumed this role on the team. The director's commitment to the program provided stability to the committee and helped the project keep to its timelines. Within another system, a clinic medical director participated in outreach and education of staff and providers. The director of psychiatry services was also active in moving sustainability efforts to the highest levels of the system. This included engaging the boards and foundations of which he was a part as well as by speaking with other physician peers to garner their support for the changes.

*Multi-disciplinary teams help increase buy-in for new processes and procedures.* All systems established multi-disciplinary teams across departments and functions to ensure system-wide buy-in for new processes. For example, one site had a team that consisted of clinic managers, cessation counselors, a medical director, director of pharmacy, information technology (IT) specialist, marketing specialist, accountant, quality program manager, vice president of operations and center director. Another health system pointed out that “It’s not just one person’s program. It’s everybody’s program. Everyone needs to have a say and have their ideas valued and incorporated”. Three of the health systems asked medical providers and staff to take part in the development and piloting of the processes and procedures they would be charged with implementing. In one system, a medical assistant took the initiative to learn the new workflow and discussed it at staff meetings. She conducted brief trainings with other staff and served as a resource for them if they had questions. This approach proved to be important in gaining buy-in and developing procedures that fit the unique workflows of specific departments, clinics or hospitals. Perhaps more importantly, multi-disciplinary involvement opened the door to additional streamlining of new procedures across “siloed” segments of the health system that do not always have compatible systems or procedures. For example, tobacco treatment specialists housed within an outpatient clinic carried beepers so that staff at the affiliated hospital could alert the specialists when a tobacco user was admitted to the hospital and needed cessation assistance. Within this same health system, the director of pharmacy helped push new nicotine replacement therapy (NRT) protocols through to the hospital side and also helped IT staff develop the medication smart set on the clinic side.

*Establishing a minimum standard intervention while allowing for flexibility can facilitate implementation.* Implementing standardized workflows or procedures across entire health systems, clinics and departments can be challenging due to differences in how entities operate. One successful technique was to create a minimum standard intervention, based on the *United States Public Health Service Guideline, Treating Tobacco Use and Dependence – 2008 Update*, that each department or clinic could implement in a manner best suited to their specific environment. For example, one system formulated a standard minimum intervention that included screening every patient for tobacco use at every visit, assessing willingness to quit and providing treatment referrals. Each clinic within that system established its own standard work processes to provide the minimum intervention as well as any additional elements (e.g., referral to on-site counseling). The project manager for that system explained, “We had ideas of the definite pieces we wanted in the [workflow], but we left space for each clinic to individualize how it might work best for them”. More specifically, at all clinics within the system either a medical assistant or nurse (“roomer”) would ask about tobacco use and assess willingness to quit at every visit and document both. At this point, a referral was either done by the roomer or by the attending physician. Some physicians preferred to conduct a brief intervention themselves and discuss medications and then refer the client to the state quitline via fax referral. Two of the clinics had on-site counseling available through a clinical pharmacist trained in tobacco cessation and one of these two clinics also had a healthy lifestyle clinic once a week that addressed tobacco use. After the clinics received feedback from the quitline fax referral triage center with a patient’s documented outcome (e.g., enrolled in quitline service), the patient was sent one of two letters: a congratulatory letter if they chose to enroll or a letter offering further assistance to those that were not reached or elected not to enroll. A standard work document was created for all four clinics to clarify staff roles.

### Use technology

*Using EHRs can facilitate implementation and documentation of tobacco treatment protocols.* EHR systems helped streamline tobacco screening, treatment and referral procedures and improved documentation in three systems. For example, one health system developed and implemented new drop-down lists or “order sets” for tobacco cessation within its EHR to allow clinicians to tailor medications to individual tobacco users. The new order sets also allow clinicians to assess and refer patients to internal or external treatment options (e.g., on-site counseling or telephone quitline) and send prescriptions to pharmacies electronically. Integrating these procedures into the EHR also helped facilitate documentation of tobacco treatment efforts. In another system, IT staff constructed a new registration process for patients and created a new ordering procedure for NRT within the EHR. The IT team created a “smart set” that allowed counselors to document how tobacco use was addressed during the clinic visit. This smart set was also used to extract data for quality improvement efforts. The IT team also created other data reports that allowed for tracking of provider’s efforts to identify and intervene with patients who use tobacco (e.g. referrals made, medications prescribed) as well as to look at patient outcomes. Another system modified their EHR so that all departments within their clinics (e.g. medical, ophthalmology, chiropractic, behavioral health) were able to electronically refer patients to on-site tobacco dependence treatment. The remaining systems found fields within their existing EHRs to document tobacco use status. These systems provided training to staff around assessing readiness to quit and providing referrals within the current capabilities of each clinic’s EHR.

*EHR limitations may exist, but these can be managed to keep systems changes moving forward.* While using EHRs helped facilitate implementation of new protocols or workflows, they also presented challenges for some health systems. Some EHRs, for example, did not have the ability to document and track treatment referrals, and therefore were not designed to support evidence-based tobacco dependence treatment. In other instances, new EHR systems were being implemented on a schedule that differed from the funding period. The project manager at that system advised others to, “respect the limits of the [EHR] but don’t let the [EHR] keep you from implementing a process”. When changes to the EHR could not be easily made, processes were created to take advantage of the strengths and to acknowledge limitations of the existing system. One system worked with IT staff and technical assistance providers to influence their future EHR system while simultaneously building new processes for screening, treatment and referral within their current system.

### Establish ongoing training and continuous quality improvement mechanisms

*Ongoing training helps maintain strong processes for treating tobacco dependence.* Implementation of new protocols involves ongoing training of staff and clinicians. All of the health systems reported conducting multiple trainings with staff and providers to ensure correct and consistent implementation of tobacco treatment protocols. Training took multiple forms, from large group to one on one. While the technical assistance providers conducted one or two trainings of staff and providers within each system, the systems change managers, clinic managers and champions conducted much of the training or retraining in groups or one-on-one. In one system, while little training had yet occurred, it appeared that future training on new protocols and EHR elements would be handled by the system’s professional education department. Another system video-recorded a training conducted by one of the technical assistance providers that was then to be made available on the system’s intranet for nurses to obtain CEUs. Peer-led training (i.e., clinician champions training other clinicians) was often better received than training provided by external staff. Given the regularity of staff turnover, the systems added tobacco intervention protocols as part of new employee orientation, and some systems included ongoing training as part of continuing quality improvement or performance-monitoring efforts. One project manager shared, “We wrote in the grant initially that we were going to do one-time trainings. We realized as the process was unfolding that we had to keep going back and re-training”.

*Using data for continuous quality improvement and performance monitoring can help identify problems and prompt actions to improve consistency of workflow processes.* All systems established monitoring procedures to ensure that new protocols were put into practice appropriately. Additionally, three systems built and implemented data extraction and reporting capabilities into their EHR to be able to monitor whether protocols were being followed. These health systems worked with quality improvement and IT staff to establish discrete data points that could be extracted from the EHR to monitor tobacco treatment delivery. This information was shared with staff and clinicians on a monthly or quarterly basis and used to identify where additional training was needed. One system used “huddle boards” in the clinic for providers, nursing staff and leaders to review and discuss different metrics such as the number of tobacco users being referred to cessation treatment. During these “huddles”, staff would brainstorm solutions to problems, such as a lack of referrals, and occasionally provided refreshers on protocols. The project manager at that clinic explained, “I can use those [huddle board] meetings as a reminder: ‘How’s the process going? . . . Are there any concerns?’ . . . It was also a place where we could report data and set some goals, such as a goal for the number of referrals per month”. Data suggest the systems will continue to monitor tobacco use and tobacco treatment protocols, at least until the procedures appear to have become routine practice for staff and providers.

### Leverage external funding and technical assistance

*External resources and assistance from technical experts can help elevate the priority of systems changes.* Almost all systems noted that changes would likely not have occurred without the targeted funding provided in this initiative, as this helped elevate the priority of tobacco-related systems changes. One system found the funding instrumental in making necessary EHR changes: “Funding really contributed quite a bit. . . . Take, for instance, the EMR changes or report writing we did within EPIC. EPIC and reporting staff have about 100 jobs to do. Without the ability to say ‘we have some funding to buy some of your [IT] staff time,’ our project would have been at or near 100. Instead, we were up in the top 10”. Another project manager shared that “the grant helped support the process, solidify commitment and helped elevate work to a higher priority”. Additionally, the availability of technical assistance allowed most systems to identify and address specific assets and gaps within their health system. While one system struggled with defining the role of the technical assistance provider, the other systems found their experiences, resources and recommendations to be instrumental in advancing their individual systems change activities. For example, one project manager reported using the technical experts with their training activities: “They came here to present an in-service to the cardiology staff. We videotaped it, put it on the learning portal and it can now be accessed by nursing staff to receive continuing education credits”. Other systems found value in resource sharing, facilitation of dialogues with other health systems undertaking this work and advice on engaging other parts of large health systems.

### Challenges and barriers for health systems

While the focus of this article is on positive facilitators of change, the four health systems also had to overcome some challenges and barriers. Some systems were forced to balance multiple system priorities while maintaining momentum and addressing staff turnover. Larger health systems had to address differences in cultures, process and electronic systems among various departments and clinics. When utilizing technology to support systems changes, some were challenged by inadequate reporting and data fields within their current EHR system. Finally, some systems had to overcome clinician reluctance to refer patients to external treatment options (e.g. quitlines). Some clinicians were also less confident in their ability to intervene with tobacco users and were concerned about the amount of time it would take to provide tobacco dependence treatment while also implementing new processes and responsibilities.

## Discussion

Findings from this qualitative analysis suggest that health systems can successfully implement substantial changes to facilitate tobacco dependence treatment in a relatively short timeframe. This study demonstrated that health systems could systematize identification and documentation of tobacco users, implement follow-up procedures and use EHR systems to facilitate systems changes. Factors positively impacting their ability to do this were capitalizing on environmental changes, ensuring leadership support, using technology, establishing mechanisms for continuous quality improvement and leveraging external funding and technical assistance.

These findings are consistent with existing literature, which shows the importance of advocacy from internal champions to successfully complete the work [14] and implementing process improvements with support from leadership [18,19]. Additionally, health systems change implementation guides highlight EHRs as tools for achieving meaningful use goals, supporting new processes and newly-trained clinical staff, and enabling efficient tobacco user documentation and intervention via a care coordination model. One new implementation guide is titled “Help Your Patients Quit Tobacco Use: An Implementation Guide for Community Health Centers [22]”. Findings from this study are consistent with these recommendations.

Furthermore, this study builds on the technology literature [17] by demonstrating the importance of using EHRs to fully integrate treatment into ongoing care delivery as well as to improve the delivery of tobacco cessation services. Using EHRs effectively will be essential for many reasons, including using them to improve delivery of care and to comply with meaningful use incentives and other federal requirements.

Health systems change initiatives in treating tobacco use have increasingly become priorities for national and state level funders. The U.S. Centers for Disease Control and Prevention’s (CDC’s) newly updated Best Practices for Comprehensive Tobacco Control Programs identifies health systems change as a key cessation activity. CDC now recommends that the focus of state-level tobacco control programs “should remain on population-level, strategic efforts to reconfigure policies and systems in ways that normalize quitting and that institutionalize tobacco use screening and intervention within medical care” [8]. The findings from this study can serve as a resource for both states and health systems likely to implement similar systems changes.

### Insights for health systems

Process improvements such as those undertaken within these health systems can be individualized and implemented in a way that works within existing systems and workflows. With technical assistance and examples of best practice protocols, health systems can improve their delivery of tobacco dependence treatment at the systems level. These improvements have potential to decrease tobacco use and improve health for their entire patient population.

Additionally, these findings highlight factors health systems need to account for in order to quickly design and implement changes for integrating tobacco treatment into routine care. The systems focused on multiple factors, such as ensuring buy-in from organizational leadership, creating a multi-disciplinary project team, using electronic health record systems as change agents and ensuring continuous training and quality improvement feedback for clinicians and staff. Addressing these factors simultaneously allowed systems to efficiently implement new processes and procedures to advance this work.

### Insights for funders

Implementing health systems changes for tobacco treatment is likely new work for funders and health systems. In undertaking this funding initiative, ClearWay Minnesota created processes to facilitate information sharing through annual site visits, all-grantee meetings and facilitated conference calls. Flexibility was also built into grant requirements, budgets and workplan design. For example, while ClearWay Minnesota required strategies to identify and document tobacco users, health systems could choose other strategies based on gaps identified during a system-wide standardized assessment (Table 2). This approach allowed ClearWay Minnesota to develop a unique understanding of health systems change that it will use in planning future funding initiatives. Other funders may wish to consider a similar approach.

Technical assistance was also important. ClearWay Minnesota contracted for technical assistance to support the grantees’ work, which allowed for provision of specific expertise in health systems change that was not available internally. The technical assistance provider and funder worked together to help grantees identify areas where they could potentially benefit from additional support throughout the funding period. It was important for grantees to have a clear understanding of the resources, tools and expertise available to them, as well as how to access those resources.

There are also possibilities for future inquiry. As health care reforms are implemented in the United States, there will be opportunities to evaluate how new health care delivery models and payment reforms facilitate or hinder integration of tobacco treatment into routine care, and the impact of treatment integration on population health outcomes intended by these new models. Additionally, there may also be interest in evaluating the effects of systems changes on both outcomes (e.g., quit rates) and patients' acceptability of and satisfaction with these types of changes and the associated treatment delivery improvements.

### Limitations

This report is subject to several limitations. This study was observational in nature, and therefore we cannot be certain whether changes occurred solely as a result of the funding initiative. This study was primarily designed to gather information that would help ClearWay Minnesota make decisions around funding of similar efforts in the future. It was not designed to measure sustainability of changes in the long term (only the *potential for* long-term sustainability), nor was it designed to measure impacts on patients as a result of these efforts (e.g., successes in quitting tobacco use). While direct, on-site observation of systems changes (such as following patients through the tobacco use identification and brief intervention processes) would have allowed for monitoring of actual implementation of systems changes, this was not possible given resources, burden on the health systems, and patient privacy and confidentiality concerns. Additionally, the small number of health systems funded, small number of interviewees from each health system, and the fact that all were located in Minnesota limit the generalizability of findings. However, many of the themes identified in this analysis (e.g., need for champions, multi-disciplinary teams, leveraging technology) have been cited both in the United States and elsewhere as necessary for supporting health systems change [23-25]. The relationship between the health systems, technical assistants, evaluators and funder is a potential source of bias; however, this risk was mitigated by using an independent evaluator and basing the evaluation on the improvement of processes rather than outcomes. Finally, ClearWay Minnesota had a long history of working with these funded projects prior to this initiative. These systems already recognized tobacco use as a priority, which may have led to more rapid implementation of systems-level changes.

## Conclusions

Health system change strategies present a significant opportunity to integrate evidence-based practices for treating tobacco dependence into routine health care. Such work is increasingly important given the implementation of Meaningful Use and other healthcare quality measures. Routine delivery of tobacco dependence treatment will become increasingly important as providers, employers, insurers and government entities look to improve the public’s health and reduce the total cost of health care. Funding for health systems change initiatives elevates the priority of this work in environments with many competing demands. Funders and health systems should consider capitalizing on the changing health care environment by investing in health systems changes for tobacco dependence treatment as one strategy to help drive down the premature morbidity and mortality associated with tobacco use.
